# Differential Diagnosis: Hepatic Complications in Inborn Errors of Immunity

**DOI:** 10.3390/jcm12237480

**Published:** 2023-12-03

**Authors:** Emily Zinser, Ky-Lyn Tan, Da-In S. Kim, Rachael O’Brien, Alison Winstanley, Patrick F. K. Yong

**Affiliations:** 1 Department of Allergy and Clinical Immunology, Frimley Health NHS Foundation Trust, Surrey GU16 7UJ, UK; 2 Department of Allergy and Clinical Immunology, Royal Surrey NHS Foundation Trust, Surrey GU2 7XX, UK; 3 Department of Cellular Pathology, University College London Hospitals NHS Foundation Trust, London NW1 2BU, UK

**Keywords:** inborn errors of immunity, primary immunodeficiency, nodular regenerative hyperplasia, CVID, hepatic infections, autoimmune hepatitis, granulomas, DILI

## Abstract

Inborn errors of immunity (IEIs) are a heterogeneous group of diverse clinical and genetic phenotypes that have an estimated combined prevalence as high as 1/1000. Increased risk of frequent, severe, or opportunistic infections is a common feature of IEIs, but there are also diverse immune-mediated, non-infective complications that are associated with significant morbidity and mortality. As patient survival increases, these are becoming more apparent within the liver. Hepatic involvement of IEIs may not only manifest as infections, but also nodular regenerative hyperplasia, granulomatous disease, autoimmune hepatitis and malignancy. As therapeutic options for patients are expanding, with both pharmaceutical treatments as well as haematopoietic stem cell transplant (HSCT), iatrogenic liver injury is increasingly common and important to identify. This review article summarises the spectrum of hepatic complications seen in IEIs, and highlights the challenges of management within this patient cohort, where immunosuppression is poorly tolerated. Early recognition and prompt diagnosis of potential hepatic complications is therefore crucial in ensuring potentially reversible causes are treated, but significant uncertainty remains regarding best practice for many features of immune dysregulation with limited high-quality evidence.

## 1. Introduction

There are currently 485 inborn errors of immunity (IEI) according to the 2022 update of the IUIS classification [1], a number that has increased year on year. Advances in genetic sequencing methods and analysis have accelerated the discovery of novel IEIs. Survival of patients with IEI is also improving [2,3], owing to a combination of developments in targeted immunomodulatory therapies, improvements in haematopoietic stem cell transplantation (HSCT) [4] and gene therapy [5] as well as the introduction of newborn screening programmes for severe combined immune deficiency (SCID) in several countries [6,7].

**Figure 1:**
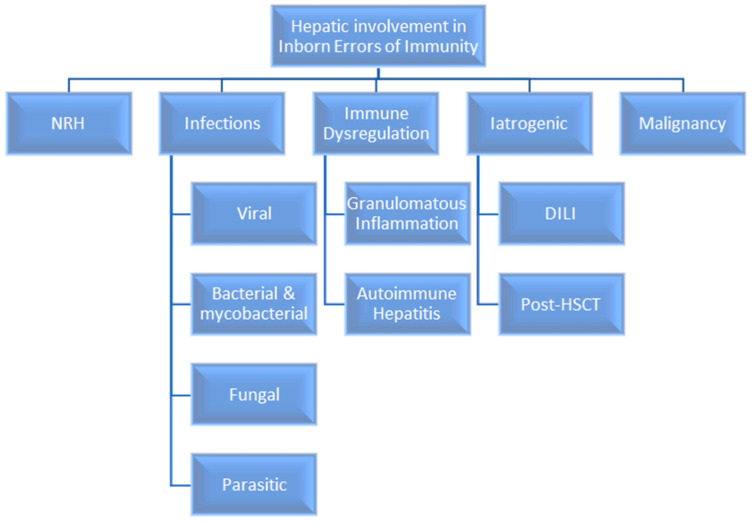
The spectrum of hepatic involvement in IEIs. NRH—nodular regenerative hyperplasia; DILI—drug-induced liver injury; HSCT—haematopoietic stem cell transplantation.

Hepatic involvement may be a hallmark for some IEI, as seen in chronic granulomatous disease (CGD) with Staphylococcal liver abscesses. However, it is well recognised that whilst IEIs predispose to a diverse array of infections, there is significant morbidity and mortality from co-existent immune dysregulation, lymphoproliferation, autoimmunity and malignancy, all of which can affect the liver (Figure 1) [8,9]. There is increasing acceptance of the likely under-recognition of nodular regenerative hyperplasia in IEI patients, especially within the common variable immunodeficiency (CVID) cohort [8,10]. Furthermore, as treatment options increase with the greater use of targeted immunomodulation, antimicrobials, and HSCT, the scope of iatrogenic hepatic injury is also increasing. This review aims to summarise the spectrum of hepatic pathology seen in many IEIs, the current gaps in our understanding and where further research is needed.

## 2. Nodular Regenerative Hyperplasia

Nodular regenerative hyperplasia (NRH) of the liver is characterised by transformation of the organised hepatic architecture into nodules, with minimal associated fibrosis and leads to noncirrhotic portal hypertension (NCPH) with complications of splenomegaly, varices and ascites. NRH was first reported in association with Felty’s syndrome [11], but has since been documented in multiple autoimmune rheumatic conditions [12,13], HIV [14,15] haematological malignancies [16], gastrointestinal diseases including inflammatory bowel disease [17], coeliac disease [18,19] and as a complication of both solid organ transplant [20,21] and HSCT [22]. It has also been reported as a drug-associated phenomenon, commonly with thioguanine, antiretroviral therapy and chemotherapeutic agents [23,24].

Within the setting of IEIs, NRH is most associated with CVID, but has been reported in other primary antibody deficiencies including X-linked agammaglobulinaemia (XLA). NRH was found in 75% (6/8) of those who underwent a liver biopsy in one XLA cohort [25]. It has also been described in genetically diverse IEIs including ataxia telangiectasia [26], NFKB1 haploinsufficiency [27], ADA2 deficiency [28], CD40 ligand deficiency [29], activated PI3K delta syndrome [30], STAT3 gain of function [31] and chronic granulomatous disease (CGD) [32]. In the CVID cohort, the prevalence of liver disease from all aetiologies is reported between 12.7% [33] and 79% [8], with the significant variability at least partly owing to different investigations undertaken between studies. The prevalence of NRH in CVID is also uncertain, with figures between 5% [34] and 32% [8]; however, Ward et al. reported NRH in 13/16 (82%) of CVID patients with either a previous liver biopsy, or abnormal liver function tests for at least 6 months [10].

The presence of liver disease is important to identify as hepatic involvement is associated with other complications, e.g., enteropathy, lymphoproliferation and autoimmunity [10,34,35,36]. Liver involvement is also linked with a significant increase in mortality. In one cohort study of 411 patients in the United States, the presence of liver disease was found to be associated with reduced survival (hazard ratio 2.48) [9]. A UK study of CVID patients corroborated this with a hazard ratio of 3.5, and a median survival of 8 years from CVID diagnosis to death in those with associated liver disease [8].

### 2.1. Pathophysiology

NRH is thought to arise from abnormalities in hepatic perfusion at the microvascular level, with functional or structural hypoperfusion within the hepatic bed leading to cellular injury, apoptosis and atrophy. Subsequent localised compensatory hyper-perfusion to adjacent acini is postulated to result in adaptive hyperplasia and generation of nodules [23]. Histopathological studies have corroborated a vascular component to NRH pathogenesis, with studies demonstrating microvascular changes with obliteration of portal venules [37,38].

NRH appears to be a final common pathway of heterogeneous clinical conditions, and it is thought that the aetiology of the disruption in perfusion varies depending on the underlying associated disease [23]. In the context of immunodeficiency, chronic antigen exposure via the gut and portal vein with subsequent inflammatory changes has been proposed as a factor in the development of NRH and NCPH. Animal studies have demonstrated the development of portal fibrosis and hypertension with splenic extract in Freund’s complete adjuvant [39], as well as E. coli via the portal tract [40]. Intrasinusoidal CD3+/CD8+ lymphocytic infiltrates have been documented in multiple histological studies of IEI patients [29,41,42,43] and are thought to mediate direct damage and apoptosis of endothelial cells, resulting in localised microvascular impairment and subsequent fibrotic response [29]. Although endotoxinaemia has been variably reported in CVID patients [44,45], they do have an altered cytokine signature compared to healthy controls, with associated T-cell exhaustion, thought to be owing to chronic gut bacterial translocation [44,46].

### 2.2. Histological Features

**Figure 2:**
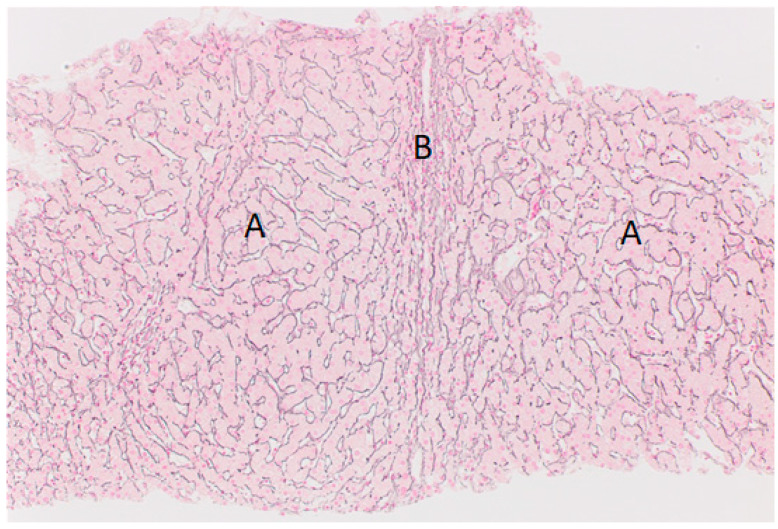
A liver core biopsy from a 37-year-old patient with CVID. This reticulin stain shows areas of thickened hepatocyte plates with nodularity (A), with a central area of compressed hepatic parenchyma (B). There is minimal associated fibrosis.

Liver biopsy is essential for the diagnosis of NRH, and the histological features (Figure 2) can be subtle, particularly in small needle core biopsies [47], and on routine use of haematoxylin-eosin stains [48]. Atrophic regions are seen adjacent to hyperplastic nodules with no intervening fibrosis, best visualised with reticulin stains [48]. Fibrosis is usually sparse, unlike cirrhosis, although periportal and perisinusoidal fibrosis may be seen in atrophic areas [48]. Although NRH is defined by absent/minimal fibrosis, studies in CVID patients have reported a high prevalence of fibrosis, as well as a sinusoidal lymphocytic infiltrate [34,41,42], with the term NRH-like changes (NRH-LC) being used [42]. As NRH is seen in association with diverse clinical conditions, its aetiology is likely multifactorial, and further work is needed in determining how NRH in association with IEIs differs histopathologically.

### 2.3. Presentation and Diagnosis

NRH remains a histopathological diagnosis confirmed by biopsy, but diagnosis of NRH in the IEI cohort can present with extra challenges. Co-morbid conditions may increase the risk of complications, e.g., immune thrombocytopenic purpura (ITP) with increased bleeding risk, and risk of infection. Furthermore, the presence of splenomegaly may be due to either portal hypertension or the underlying IEI and is not necessarily a reliable marker for NCPH. Similarly, abnormalities in liver enzyme markers may be multifactorial, including drug-induced-liver-injury (DILI), infection and other common non-immunological conditions including non-alcoholic fatty liver disease (NAFLD) and non-alcoholic steatohepatitis (NASH). Transient elastography (TE) has become an increasingly useful non-invasive technique to highlight potential hepatic involvement and which patients may benefit from biopsies. Crescenzi et al. demonstrated elevated liver stiffness of at least moderate fibrosis in 33.8% of their CVID cohort, and that liver stiffness correlated with serum alkaline phosphatase (ALP), gamma glutamyl-transferase (GGT), longitudinal spleen length but not serum aspartate aminotransferase (AST)/alanine transaminase (ALT) [36].

The presenting features of NRH in IEIs are highly variable and range from asymptomatic to complications of portal hypertension with variceal bleeds, splenomegaly and ascites. Abnormal hepatic enzymes are seen in a large proportion with an isolated elevated ALP being a common and relatively early finding [10,34,36]. ALP abnormalities vary and can include progressive elevation, or fluctuations either persistently or intermittently outside of the normal range [10]. Abnormal AST and ALT, as well as deranged synthetic function are also seen [10,34]. Importantly, NRH has been described in association with normal hepatic enzymes [10], highlighting the importance of having a low threshold to undertake other investigations such as TE in selected patients. Interestingly, Azzu et al. reported NRH-like changes with histological cirrhosis in a subgroup of patients [8], blurring the classical distinction that NRH is associated only with NCPH, and clinically corroborating the histological studies that have demonstrated overlap between NRH and fibrosis in the IEI and CVID cohorts.

Owing to small numbers of patients diagnosed early and followed up, the natural history and progression to portal hypertension with associated complications is uncertain. In case series, not all patients with NRH have established complications of liver disease at the time of publication, whereas a significant proportion have already progressed to jaundice, ascites and varices [8,10,34]. Sequential biopsies are rarely undertaken in patients, but in seven patients where serial biopsies were available (median 50 months between sampling), non-progression was present in two out of seven patients [41]. Further work to characterise the differences between patients that have non-progressive histological changes to those that progress to hepatic failure is crucial if we are to alter the natural disease course.

### 2.4. Treatment

There are no current high-level evidence-based treatments available for NRH, with management focusing on identification of the underlying aetiology, where applicable, and modification of other potential hepatic insults with lifestyle modifications. Screening for complications of portal hypertension is important, and will include regular endoscopic assessment for varices, and ultrasonography for spleen size. Low dose budesonide has been used in one patient with CVID, NRH and enteropathy, with improvement in ALP and GGT [49]. There has been a recent case report of the successful use of rituximab in a patient with a heterozygous transmembrane activator and calcium modulator and cyclophilin ligand interactor (TACI) mutation with splenomegaly, granulomatous lymphocytic interstitial lung disease (GLILD), ITP and NRH with NCPH. Rituximab (for ITP), with a short 4-day course of dexamethasone was associated with resolution of ascites up to 5 years after treatment, although re-assessment of hepatic investigations was not undertaken [50]. Similar to patients with end-stage liver disease from all causes, orthotopic liver transplant (OLT) has been explored in the IEI cohort. However, this has come with additional complications in that IEI patients are at much higher risk of complicated and atypical infections in the context of immunosuppression. One case series of four patients with CVID and NRH who underwent OLT reported infective complications including PJP pneumonia, C. difficile diarrhoea, toxoplasmosis, influenza pneumonia, cytomegalovirus (CMV) viraemia and proctitis and invasive pulmonary and neuroaspergillosis [51]. Non-infective complications were also noted with tonsillar squamous cell carcinoma and acute myeloid leukaemia. Disease recurrence was also high in 75% of patients. Early NRH-like changes have been reported as early as 5 months post-transplant in one case, with others demonstrating recurrence at 12- and 18-months post-transplant [51]. The 3–5-year survival of CVID patients receiving OLT is 52% with viral hepatitis excluded as a cause of hepatic failure, compared to 89% 3-year survival for all-causes of transplantation [51].

Although most reported in CVID, OLT has been reported in patients with XLA, also with variable outcomes. One patient with XLA and hepatitis C in a Norwegian cohort underwent OLT and died from disseminated C. parvum infection [52], with 2 XLA patients in a recent US registry alive post-OLT [53].

There has been increasing discussion about the role of HSCT in genetically undefined IEI, including CVID. In the context of solid organ transplantation (SOT), combined HSCT and SOT would theoretically correct the underlying immunological defect, allow appropriate immunosuppression to prevent rejection, reducing risk of disease recurrence, whilst mitigating the high risk of complicated and opportunistic infections. Mortality in HSCT for CVID is significant, with one study reporting 52% (13/25 patients), and higher in patients with hepatic involvement [54]. Although this cohort included 21 patients transplanted before 2000 there are still variable outcomes reported in CVID patients undergoing HSCT [54]. In patients with NRH, it is also uncertain as to whether correction of the underlying immunological defect prevents progression of hepatic disease.

For NRH and IEIs, there are huge numbers of uncertainties; how NRH in IEIs differs from NRH from other causes, what the natural disease course is, why some patients appear to have a more aggressive clinical pathway to NCPH and fibrosis, and what can be done to treat NRH or slow disease progression. Further work internationally is urgently needed to elucidate these if we are to address this increasing complication.

## 3. Hepatic Infections in IEI

Inborn errors of immunity (IEI) are characterised by a greater incidence and severity of infections. A significant challenge in identifying the infective pathogen is the frequent false-negative serology testing due to defects in specific antibody production. Patients receiving immunoglobulin replacement therapy may also have false-positive serology, meaning direct methods of detecting the microorganism via cultures, polymerase chain reaction (PCR) testing or microscopy may be required for an accurate diagnosis [55]. Whilst we focus on hepatic infections occurring in IEI, many gastrointestinal infections have concurrent hepatic involvement, as seen with cryptosporidium, so appropriate gastrointestinal investigations may also need to be undertaken. Table 1 shows the spectrum of hepatic infections seen in IEI.

### 3.1. Viral Infections

Viral infections are a frequent complication of IEIs; whilst some viruses are hepatotropic (e.g., Hepatitis B and C), others such as EBV have broader tropism and liver involvement may present as only one part of the clinical picture.

XLA is associated with increased susceptibility to infection with enteroviruses, such as poliovirus, coxsackievirus and echovirus, which can cause hepatitis and chronic meningoencephalitis [59].

Hepaciviruses include hepatitis C virus, the second commonest cause of chronic viral hepatitis world-wide [78]. Hepatitis C infection has arisen from contaminated blood products, including IVIg historically, causing a significant number of iatrogenic cases in CVID and XLA patients [60]. Those with inborn errors of immunity have had significantly higher morbidity and mortality reported compared with other cases of iatrogenic hepatitis C, with a UK cohort demonstrating significant 5-year morbidity with 24% reaching end-stage liver disease and mortality rates of 32% [79]. As these cases were published prior to the use of direct-acting anti-virals (DAAs), treatment including interferon monotherapy with small numbers of patients also receiving ribavirin was trialled, with limited success, and some patients ultimately required OLT [79].

As expected with the severe underlying immunodeficiency, multiple different viruses have been reported in association with severe combined immunodeficiency (SCID), including hepatitis from adenovirus, cytomegalovirus and varicella zoster virus. Severe manifestations and complications of fulminant hepatitis and hepatic necrosis have been described [56].

Viral hepatitis from CMV, EBV, Hepatitis B and C have been reported in patients with DOCK8 deficiency [57]. CMV-associated hepatitis is the most frequent cause of viral hepatitis in MHC Class II deficiency [58].

Hepatitis E virus appears to have a lower prevalence with fewer documented reports within inborn errors of immunity, which may be associated with the neutralising antibody in many infused immunoglobulin products [80]. There has been a recent case report of CVID patient with acute hepatitis owing to hepatitis E, which highlights the importance of maintaining vigilance even in those established on immunoglobulin replacement [81].

Epstein–Barr virus (EBV) is a gamma herpesvirus that affects 95% of the population. EBV-susceptible IEIs are seen with mutations in SH2D1A, XIAP, CD27, CD70, CTPS1, TNFRSF9, MAGT1, RASGRP1, TET2 and CARMIL2 [1]. Hepatic dysfunction and hepatosplenomegaly can be seen as part of the clinical picture in a number of IEIs associated with EBV susceptibility. The clinical features of these conditions vary significantly, and range from fulminant haemophagocytic lymphohistiocytosis (HLH), EBV-driven malignancies, as well as immune dysregulation with a lymphoproliferative syndrome with associated liver dysfunction, hepatosplenomegaly, hypogammaglobulinaemia and lymphadenopathy [61].

Several other IEIs have also reported chronic active EBV disease as a component of a broader combined immunodeficiency, including mutations in GATA2, PIK3CD, COPG1, HELIOS, AIOLOS, IL2RB, STK4 and ITK [1,82].

### 3.2. Bacterial Infections

Bacterial hepatic infection can present as either abscesses, or cholangitis. Chronic granulomatous disease (CGD) is an IEI with impairment of the phagocyte respiratory burst, causing susceptibility to catalase-positive bacteria and fungi. Hepatic abscesses are a frequent presenting feature and complication, occurring in up to 35% of patients, and are the commonest cause of deranged liver function in CGD [83]. *Staphylococcus aureus*, *Pseudomonas aeruginosa* and *Burkholderia cepacia* are the most common causative pathogens, and are reported in 25–45% of CGD patients with high mortality rates of up to 27% [62,63]. Liver abscesses are challenging to treat, with a high incidence of recurrence [84]. Abscesses are often complex, septated and surrounded by a thick pseudocapsule, rendering treatment more challenging; prolonged antibiotic courses are required, and the addition of steroids has been shown to reduce the need for repeated interventions [84]. Further treatment options may be required including surgical excision or radiological-guided drainage.

Although Staphylococcal abscesses are more commonly seen in the skin and lungs in autosomal dominant hyper IgE syndrome, liver abscesses are reported [64]. There are case reports of Staphylococcal abscesses associated with interleukin-1 receptor activated kinase 4 (IRAK4) deficiency [65,66].

Bacterial cholangitis associated with pseudomonas, enterococcus and streptococcus infections have been seen in MHC Class II deficiency [58].

### 3.3. Mycobacterial Infections

Non-tuberculous and tuberculous mycobacteria can have a broad presentation with varied organ involvement and are seen in many IEIs including (and not limited to) SCID, CID, Mendelian Susceptibility to Mycobacterial Disease (MSMD), CGD, GATA2 deficiency, TYK2 deficiency and defects in NF-KB signalling [69]. Hepatic involvement can arise as a specific complication of mycobacterial disease; vaccine-associated disease after BCG inoculation has been reported to affect the liver in 15% of BCG-vaccinated SCID cases in an international survey [67]. BCG-osis is also a known complication in Mendelian Susceptibility to Mycobacterial Disease; in one Mexican cohort, 21% (3/14) of children with IL-12Rβ1 deficiency developed hepatic BCG-osis, with one patient developing portal hypertension owing to portal vein compression from adenopathy [68].

### 3.4. Parasites

Cryptosporidium is a water-borne protozoa that can cause prolonged infections with significant morbidity and mortality within the immunocompromised. Up to one third of patients with CD40L deficiency have liver involvement [85], with Cryptosporidium-related gastrointestinal and/or biliary tract disease affecting between 6 and 21% of CD40 ligand deficiency patients [3,70]. Chronic Cryptosporidium infection is associated with secondary sclerosing cholangitis which can significantly increase mortality [3,70]. Although a proportion of patients with sclerosing cholangitis do not have detectable Cryptosporidium, this may relate to assay sensitivity [85]. Cryptosporidium is a well-recognised complication in the phenocopy hyper-IgM syndrome, autosomal recessive CD40 deficiency [86,87]. Cryptosporidium has also been reported in PI3 kinase disease (from GOF mutations in PIK3CD or loss of function mutations in PIK3R1) [71], IL-21 receptor deficiency [72], idiopathic CD4 lymphopaenia [73], DOCK8 deficiency [74] and MHC Class II deficiency [58].

There has been extra-intestinal localisation of *Giardia lamblia* within the liver in CVID patients where giardiasis is a common cause of chronic enteritis [75]. Alveolar echinococcus with associated hepatic failure has been reported in a patient with AD-HIES [88].

### 3.5. Fungi

Given the susceptibility to Aspergillus in view of the underlying immunodeficiency, invasive Aspergillosis with hepatic involvement, as well as hepatic abscesses, have both been reported in association with CGD [76,77]. Extra-pulmonary infection is most commonly seen with Aspergillus fumigatus [89]. Candida has also been reported, although is a much rarer cause of hepatic abscesses in CGD (2% of 98 cases of liver abscesses in one cohort of 368 patients) [62].

## 4. Immune Dysregulation

Autoimmune conditions are more frequently found in patients with IEI in comparison to the general population. A retrospective study showed that autoimmunity was seen in 26.2% of patients with IEIs, with CVID and combined immune deficiencies showing the highest association with autoimmune disease [90]. The presence of autoimmunity is also associated with poorer outcomes [90]. Whilst autoimmune cytopaenias are the most frequent complication, involvement of the liver is less common, with few cases reported in the literature [91]. Immune dysregulation is most commonly seen as granulomas or autoimmune hepatitis (AIH), with rare case reports of primary biliary cirrhosis (PBC) or primary sclerosing cholangitis (PSC) [85,90,92,93,94,95].

### 4.1. Pathophysiology of Immune Dysregulation in IEI

Although the association between autoimmunity and many IEIs is incompletely understood, the aetiology will vary according to the underlying immunological defect. Some IEIs and their association with autoimmunity are well characterised, as shown in Table 2; however, many IEIs, including the heterogeneous cohort of CVID patients, often have significant underlying immune dysregulation, granuloma formation and autoimmunity, which is poorly understood in terms of underlying pathogenesis, making treatment particularly challenging.

### 4.2. Granulomas

Granulomas are localised collections of a central accumulation of macrophages with a surrounding rim consisting of lymphocytes and fibroblasts. The pathophysiology behind the formation of granulomas in patients with IEI is unclear. The prevalence of granulomas in IEIs is reported between 1 and 4%, with the highest rates reported in CVID, combined immune deficiency (CID) as well as CGD [103]. Granulomatous involvement in CGD may be infectious as well as non-infectious, owing to the hyper-inflammatory state seen in this condition [104]. In CVID, several studies have shown an increased incidence of granulomas in CVID patients with autoimmunity, especially immune thrombocytopenic purpura (ITP) and autoimmune haemolytic anaemia (AIHA) [95,105,106]. In CVID, the prevalence of granulomas varies between 2 and 20% [35,107], and in one systematic review the liver was the fourth most frequently affected organ [106], other commonly affected sites being the lungs, skin and lymph nodes. Granulomas are associated with poorer outcomes in CVID patients [95,105,106].

Diagnosis is usually guided by imaging and liver biopsy, as biochemically they present non-specifically, often with raised ALP and GGT [108]. Histologically, theses granulomas are non-caseating and may be mistaken for sarcoidosis [108,109]. It is therefore important to look for other histological features that are characteristic in CVID, such as lack of plasma cells and poorly formed germinal centres [109]. It is important to rule out infective causes in this subgroup of patients.

Management is challenging as it involves immunosuppression in a group of patients who are already immunosuppressed, and therefore at high risk of complications and opportunistic infections [105,106,108]. There are no randomised controlled trials as to what therapy is best for treatment of granulomatous lesions in IEI patients. A systematic review of granulomatous disease in CVID patients describe the most common treatment modality being steroids; however, relapse is common upon cessation [106]. There is success in treating granulomas in different organs described with anti-TNF therapies, the most common being infliximab; however, the numbers are small and multi-centre randomised trials are urgently required [106].

### 4.3. Autoimmune Hepatitis

AIH is an autoimmune chronic inflammatory liver disease characterised by the presence of autoantibodies, elevated hepatocellular enzyme levels and excessive hepatic lymphoblastic infiltration in portal tracts, portal interface and parenchyma [91]. AIH in IEI can be categorised into ‘classical’ AIH associated with autoantibodies and AIH with negative serology but histology consistent with AIH. In patients with IEI, serological testing is frequently unreliable [92,93,108], whilst the use of immunoglobulin replacement makes interpretation of serological markers challenging. AIH can be seen in a variety of IEIs associated with immune dysregulation and loss of tolerance, but is a frequent complication in autoimmune polyendocrinopathy with candidiasis and ectodermal dystrophy (APECED) patients, with frequencies of APECED-associated hepatitis (APAH) as high as 42% in a North and South American cohort [110]. In CVID, patients can develop an AIH-like liver disease, with negative autoantibodies, but histological features suggestive of AIH, although there is no consensus or standardisation on diagnostic criteria. AIH-like changes have also been reported in co-existence with NRH [34], which is atypical for classical AIH. It has been proposed that this infiltrate may represent a more extreme variant of the perisinusoidal lymphocytes seen in NRH-LC in CVID and sits along of spectrum of inflammation and immune dysregulation within the liver [111].

Hepatocellular injury is seen biochemically, with raised ALT and AST, with or without the presence of autoantibodies. Liver biopsy is essential for diagnosis. There are many histological features that can be seen, but severe interface hepatitis is considered a hallmark of AIH [112]. Other features include plasma cell predominance in the portal inflammatory infiltrate and regenerative rosettes and emperipolesis [112]. The diagnosis requires a combination of clinical, biochemical and histological indices. It should be considered especially when all other known causes of liver disease have been excluded.

Management is clinically challenging with evidence on best practice limited to few case reports. Management of AIH in patients with IEI usually involves the use of corticosteroids with or without additional immunosuppressants [85,113]. Further immunosuppression in these already immunodeficient patients can prove challenging in view of infection risk. With the advances in genetics and identification of mutations, the use of targeted immunotherapy depending on the IEI should be considered [94,114]. Three out of six patients with STAT 3 GOF and AIH showed improvement in liver enzyme markers with the use of jakinibs (ruxolitinib and tofacitinib) and tocilizumab as an adjunct. However, one of these patients with multiple complex co-morbidities passed away with significant advanced disease and infection burden [114]. HSCT can also be considered in patients with refractory autoimmunity; however, there are no case reports in the literature on this subgroup of patients [114,115].

### 4.4. Primary Biliary Cholangitis

PBC is an autoimmune disease that affects the biliary tract, associated with anti-mitochondrial autoantibodies. It is rarely reported in the IEI populations; however, a case of a patient with IL-2 receptor alpha deficiency and primary biliary cirrhosis experienced disease remission post-HSCT [115].

### 4.5. Primary Sclerosing Cholangitis

PSC is a chronic cholestatic liver disease of unknown pathogenesis with fibro-obliterative sclerosis of intra- and/or extrahepatic bile ducts. Its pathogenesis remains unclear [116]. Again, rare in the IEI cohort, there is successful transplantation in a patient with CVID and PSC; however, the case report did not include long-term outcomes [117].

## 5. Iatrogenic

As the scope of therapies increases for IEI patients, iatrogenic liver disease is becoming an increasingly common complication, and can be caused by pharmacological, diagnostic or interventional measures carried out on patients. The main causes in patients with IEI can broadly be split into drug-induced and post-haematopoietic stem cell transplantation (HSCT) complications.

### 5.1. Drug-Induced Liver Injury (DILI) in IEI

DILI should be considered in all IEI patients who present with liver injury, where there is a broad spectrum of possible agents, as shown in Table 3. The most common culprit in IEI is the use of antimicrobials in prevention or treatment of infections, and it is one of the most common drug class of DILI worldwide [118,119]. Patients with IEI often require prolonged courses or higher doses of antimicrobials. Multiple studies have shown that co-amoxiclav is often the cause, other common agents are isoniazid, sulphonamides and nitrofurantoin [118,120,121]. DILI usually occurs after a latency period of days to months following exposure [120]. It can be categorised into hepatocellular injury, cholestatic or mixed, depending on their liver biochemistry abnormality. It can be further classified into immune and non-immune mediated reactions. Immune reactions are often associated with fever, rash, eosinophilia and auto-antibodies [118]. They show early onset and rapid reinjury is seen with reintroduction of the drug. Non-immune mediated reactions have a later onset of action, up to 1 year and are not associated with rapid reinjury with reintroduction of the drug [118]. A detailed drug history is needed to help identify if DILI could be a potential cause of their liver enzyme derangement. 

The diagnosis can be unclear and liver biopsy may be required. The presence of canalicular cholestasis favours DILI, whereas rosette formation, portal plasma cells, severe portal inflammation and the presence of fibrosis favours AIH [120]. HLA genotyping can be utilised to support diagnosis, e.g., HLA-B*5701 is associated with flucloxacillin-induced DILI, whereas HLA-DRB1*1501 is associated with amoxicillin/clavulanate-induced DILI [120].

Identification and subsequent withdrawal of the culprit drug is key, not only for the acute episode of DILI, but in terms of guiding future treatment in preventing further worsening of a patient’s liver function. The use of corticosteroids remains limited and is reserved for those with immune-mediated DILI or those with drug induced AIH-like picture. Liver transplantation is reserved for severe cases [118,120].

### 5.2. Post-HSCT Complications

For many IEI, HSCT is the gold standard treatment, and liver disease can be a complication of HSCT. The most common liver complication seen is hepatic veno-occlusive disease (VOD), also known as sinusoidal obstruction syndrome (SOS) and is associated with high morbidity and mortality risk. Other complications include infection, graft-versus-host disease (GvHD) and drug toxicity (see above) [130,131].

#### 5.2.1. VOD/SOS

VOD/SOS occurs due to use of high-dose chemotherapy with HSCT causing endothelial injury [126,130,131]. Despite reduced-intensity conditioning regimens, VOD/SOS remains a major issue. Its incidence has been reported to range from 8–14% with fatality rates in excess of 50% in early studies [131]. Treatment for VOD is limited and mainly supportive, with defibrotide being the only treatment licensed for VOD in the European Union. Disentangling the patient versus transplant related risk factors for VOD is challenging, but in the context of IEI, familial HLH is associated with higher rates of VOD [132]. Although incompletely understood this may be owing to treatment-related toxicity. EBMT/ESID working party guidelines suggest defibrotide prophylaxis can also be considered for Omenn’s syndrome, where endothelial insults are a common feature [133].

#### 5.2.2. Infection

Opportunistic infections need to be considered in this subgroup of patients. With regards to the liver, it is important to consider reactivation of viral hepatitis including hepatitis B, as well as non-hepatotropic viruses that can cause hepatic inflammation including CMV, which has been associated with poorer outcomes if reactivation occurs [134]. Pre-transplant viral screening is therefore important to ensure prophylactic antiviral therapy is pre-emptively started [126,130].

#### 5.2.3. GvHD

GvHD occurs due to donor immune cells attacking host tissues, and hepatic involvement can be seen in both acute and chronic GvHD. In acute GvHD, hepatic involvement is reported in up to 44% of patients, and usually occurs alongside cutaneous (70%) and gastrointestinal (74%) involvement [135]. For chronic GvHD, reported rates of hepatic involvement vary up to 31% and are associated with poorer outcomes [136]. GvHD causing liver disease presents as deranged liver biochemistry, usually manifesting as progressive or sudden elevation of ALP and GGT. Severity is staged based on bilirubin levels. Liver biopsy is often required to establish the diagnosis. Management includes steroids with or without immunosuppressants such as anti-thymocyte globulin and calcineurin inhibitors [126,137].

## 6. Hepatic Malignancy in IEI

The immune system is also crucial in the anti-tumour response, as well as defence against infections. The United States Immune Deficiency Network (USIDNET) registry has identified the prevalence of cancers within the IEI cohort to be as high as 4.7%, a 1.42-fold increased risk when compared to the incidence within the Surveillance, Epidemiology and End Results (SEER) Program database, with lymphoid malignancies having the highest recorded incidence [138]. The increased risk of non-Hodgkin’s lymphoma is seen in a broad array of IEIs, not only CVID but also combined immunodeficiencies including SCID, WAS and ATM [138]. The liver is a relatively common site for extra-nodal localization of non-Hodgkin’s lymphoma, as well as a frequent site for metastases from other solid tumours [139].

Within the USIDNET Registry, CVID patients had the highest rates of reported cancers [138]. CVID is associated with a 50-fold increased risk of gastric adenocarcinoma, and 30-fold increase in lymphomas [140]. However, the prevalence and outcome of hepatic malignancies in CVID is much less documented and are most associated with hepatitis C virus-related cirrhosis [141]. Hepatocellular carcinoma does not appear to be more frequent in CVID. There has been a recent case report of hepatic angiosarcoma in a patient with XLA and associated NRH [142].

X-linked hyper IgM syndrome carries an increased risk of malignancies involving the liver and biliary tree [143]. It is postulated that chronic cryptosporidium infection leads to bile duct dysplasia [6]. Both hepatocellular carcinoma and cholangiocarcinoma are reported, as well as pancreatic and neuroendocrine tumours [143].

## 7. Other Hepatic Complications Associated with IEIs

Hepatic complications may be caused by a broad spectrum of IEIs and conversely, an isolated IEI can result in very diverse hepatic pathologies. However, there are some rare IEIs that are associated with specific liver complications, as mentioned below.

Vasculitis involving the liver has been described in Wiskott–Aldrich syndrome, with hepatic artery aneurysm rupture and thrombosis [144,145,146]. Deficiency of ADA2 is also associated with vasculitis and can cause a variety of clinical presentations including early onset strokes, rashes, cytopaenias and immunodeficiency. Hepatic involvement with vasculitis, as well as complications such as NRH [28], elevated transaminases and portal hypertension are all reported in association with ADA2 deficiency [147,148].

Veno-occlusive disease with immunodeficiency (VODI) is a rare autosomal recessive disease owing to mutations in SP110, that presents in the first year of life. Histology demonstrates a veno-occlusive disease with central vein and perivenular sinusoidal fibrosis. Infections reflect a combined immunodeficiency, with opportunistic infections including PJP, CMV, rotavirus, Candida and further complicated with cerebrospinal leukodystrophy [149,150].

Dyskeratosis congenita (DC) is a telomere biology disorder (TBD) associated with risk of bone marrow failure with aplastic anaemia, myelodysplastic syndrome (MDS) and acute myeloid leukaemia (AML). Hepatic and pulmonary fibrosis are recognised complications [151], with a recent study also reporting NRH in three out of four biopsies of affected children in one small case series, as well as one hepatic angiosarcoma [152].

## 8. Conclusions

Hepatic involvement in IEIs is becoming increasingly recognised and reported and can range from infection requiring appropriate antimicrobials to immune dysregulation requiring immunosuppression, or withdrawal of a hepatotoxic drug. The differential diagnosis must remain broad and aggressive diagnosis of the underlying cause is critical to ensure appropriate therapy (or cessation of therapy) is initiated. When approaching the patient with IEI and hepatic dysfunction, there are multiple diagnostic pitfalls that must be considered. For infections, extended pathogen testing is often required, with an emphasis on culture and molecular identification owing to limitations in serological methods. For diagnosing NRH in the IEI cohort, CVID patients with NRH have increased rates of fibrosis, sinusoidal and perisinusoidal CD8+ T cell infiltrates, and subtle histological features that differ from the original diagnostic criteria applied by Wanless [38]. In AIH, depending on the type of IEI, seropositive or seronegative AIH can occur, with a lack of consensus on diagnosing the latter.

The heterogeneity of diagnoses within the literature makes our understanding of the prevalence, natural history and treatment of these conditions additionally challenging. Urgent international collaboration is required, particularly for randomised controlled trials to investigate the safest, most effective treatment options for these patients, especially those who lack a genetic diagnosis, where targeted immunomodulatory therapies may not be readily available or considered.

With the increase in mortality with hepatic involvement in CVID, most commonly as NRH, action is needed to identify these patients early and preferably by non-invasive methods. Further research is required to identify factors influencing the rates of progression, and therefore how to modify the natural disease course [8,9].

Finally, the presence of more common hepatic pathology independent to the presence of an IEI must always be considered. Non-alcoholic fatty liver disease has a global prevalence of 25% [153] with alcohol related liver disease (ARLD) having a global prevalence of 4.8% [154]. The differential diagnosis for a patient with IEI and hepatic dysfunction is therefore extremely broad and must encompass both rare hepatic complications associated with IEIs and therapeutics, but also common hepatic diseases. Where treatment for many of these complications such as NRH is lacking, attention must be paid to appropriate lifestyle modification and risk management to prevent other high frequency liver diseases, mitigating as many other hepatic insults as possible.

## Figures and Tables

**Table 1:** The spectrum of infective complications in IEIs that have been reported to affect the liver and/or biliary tree.

	Organism	Associated IEI
**Viruses**	CMV	SCID, DOCK8 deficiency, MHC Class II deficiency [56,57,58]
	Adenovirus	SCID [56]
	Enteroviruses (poliovirus, coxsackievirus, echovirus)	XLA [59]
	Hepatitis C	Patients receiving historical immunoglobulin replacement therapy [60]
	VZV	SCID [56]
	EBV	Mutations in SH2D1A, XIAP, TNFRSF9, CD27, CD70, CTPS1, MAGT1, RASGRP1, PRKCD, TET2, CARMIL2, GATA2, ITK, STK4 [1,61]
**Bacteria**	*Staphylococcus aureus*	CGD, AD-HIES, IRAK4 deficiency [62,63,64,65,66]
	*Pseudomonas aeruginosa*	CGD, MHC Class II deficiency [58,62,63]
	*Burkholderia cepacia*	CGD [62,63]
	*Enterococcus*	MHC Class II deficiency [58]
	*Streptococcus*	MHC Class II deficiency [58]
**Mycobacteria**	BCG	SCID, MSMD [67,68]
	NTM	SCID, CID, MSMD, GATA2 deficiency [69]
**Parasites**	*Cryptosporidium*	Mutations in CD40 ligand, CD40, DOCK8, MHC II, IL-21 receptor; PI3K disease [3,58,70,71,72,73,74]
	*Giardia lamblia*	CVID [75]
**Fungi**	*Aspergillus* sp.	CGD [76,77]
	*Candida* sp.	CGD [62]

CMV: Cytomegalovirus; SCID: Severe Combined Immunodeficiency; DOCK: Dedicator of Cytokinesis; MHC: Major Histocompatibility Complex; XLA: X-linked agammaglobulinaemia; VZV: Varicella Zoster Virus; EBV: Epstein–Barr Virus; CGD: Chronic Granulomatous Disease; AD-HIES: Autosomal dominant Hyper-IgE syndrome; BCG: Bacillus Calmette-Guerin; NTM: Non-tuberculous mycobacteria: MSMD: Mendelian Susceptibility to Mycobacterial Disease; CID: Combined immune deficiency IL-21: Interleukin-21; PI3K: PI3 Kinase; CVID: Common Variable Immunodeficiency.

**Table 2:** Summary of the pathophysiology of IEI conditions associated with autoimmunity.

Inborn Error of Immunity	Pathophysiology for Autoimmunity
**ALPS**	Failure of FAS-mediated homeostasis and apoptosis of autoreactive lymphocytes. 2/3 of patients have underlying FAS mutations [92,96]. Other mutations are reported in FAS Ligand, Caspase 8 and 10, NRAS and KRAS [97].
**APECED**	Autoimmune regulator (AIRE) gene mutations resulting in failure of central T cell tolerance [98].
**CTLA-4 Haploinsufficiency**	CTLA-4 is an inhibitory receptor expressed by activated T cells and FOXP3+ regulatory T lymphocytes (Tregs). Mutation leads to dysregulated T cell activation, with loss of Treg function [99].
**IPEX syndrome**	Mutation in transcription factor forkhead box p3, critical for development and function of T regulatory cells
**STAT1 Gain of function**	GOF mutations in STAT1 gene, with increased type 1 interferon signalling [100].
**STAT3 GOF**	Mechanism currently not fully understood, but postulated to increase Th17 differentiation, possibly with impaired Treg and Tfh development [101].
**LRBA deficiency**	Affects CTLA-4 trafficking, leading to low levels of CTLA-4 and subsequently dysregulated T cell activation, and loss of Treg function [102].
**Wiskott-Aldrich syndrome**	Loss of WAS protein, which has a key role in signalling from TCR to the cytoskeleton, resulting in quantitative and qualitative impairment of T regulatory lymphocytes [92].

Autoimmune Lymphoproliferative syndrome—ALPS, Autoimmune polyendocrinopathy with candidiasis and ectodermal dystrophy—APECED, Cytotoxic T lymphocyte antigen—CTLA, Immune dysregulation, polyendocrinopathy, X-linked—IPEX, Signal transducer and activation of transcription—STAT, Tfh—T-follicular helper cell, Lipopolysaccharide-responsive and beige-like anchor protein—LRBA, Wiskott Aldrich Syndrome—WAS.

**Table 3:** Summary of drugs commonly used in patients with IEI and its associated hepatic complications.

Indication	Type of Drug	Drug Class	Hepatic Complication
**Prophylaxis or treatment of infections**	Antibiotics	Penicillins	Cholestatic, mixed [118,120,121] (mainly amoxicillin-clavulanate)
		Fluoroquinolones	Hepatocellular, cholestatic, mixed [118]
		Sulphonamides	Cholestatic [121]
		Nitrofurantoin	Hepatocellular [118]
		Anti-tuberculosis (Mainly isoniazid, rifampicin)	Hepatocellular [118,121]
		Macrolides	Cholestatic [121]
		Tetracyclines	Hepatocellular [118]
	Antivirals	Interferon	Hepatocellular (typically transient and mild) [122]
	Antifungals	Azoles	Hepatocellular [123]
	Anti-helminthic	Benzimidazoles	Hepatocellular (typically transient and mild) [121]
**Immune dysregulation**	Immunosuppressants	JAK inhibitors	Hepatocellular (usually transient) [124]
		TNF inhibitors	Hepatocellular, cholestatic, risk of reactivation of viral hepatitis [124]
		mTOR inhibitors	Hepatocellular (typically transient), cholestatic (rare) [125]
		Anti-CD20	Hepatocellular [124]
		Calcineurin inhibitors	Cholestatic [126]
**Immunodeficiency**	Blood products	Immunoglobulin replacement therapy	Risk of viral hepatitis. Although risk is now negligible due to adequate screening [127]
**Hereditary angioedema**	Androgens	Danazol	Cholestatic, hepatocellular (usually transient). Rarely associated with hepatocellular adenoma [128] and carcinoma [129]

JAK—Janus kinase; TNF—tumour necrosis factor; mTOR—mammalian target of rapamycin.

## Data Availability

No new data were created or analyzed in this study. Data sharing is not applicable to this article.
